# Revealing hidden tissue architecture with mid-infrared dichroism photoacoustic microscopy

**DOI:** 10.1038/s41377-026-02218-4

**Published:** 2026-02-24

**Authors:** Colton McGarraugh, Soon-Woo Cho, Junjie Yao

**Affiliations:** Department of Biomedical Engineering, Duke University, Durham, NC USA

**Keywords:** Optics and photonics, Imaging and sensing

## Abstract

By combining mid-infrared excitation with polarization-resolved photoacoustics, Park et al. introduce a novel label-free approach to visualize both molecular composition and fiber alignment in engineered tissues. This dual-contrast framework, termed MIR-DS-PAM, offers a new path toward analytical, quantitative histopathology.

Biological tissues rely on precise molecular alignment to sustain mechanical and physiological function. Disruptions in this organization underlie fibrosis, infarction, and cancer^1–3^. Yet current conventional imaging techniques capable of observing these structural alignments- traditional confocal fluorescence microscopy (CFM)^4^, immunofluorescence^5^ and H&E staining^6^ - require exogenous labeling to visualize specific structures or molecules. These labeling techniques can be labor-intensive, are prone to variability, and lack longitudinal analysis. As a result, there has been a critical need and a drive for label-free techniques capable of revealing tissue architecture and composition simultaneously.

In recent years, several label-free imaging techniques have been developed for quantitative molecular mapping. Fourier-transform infrared technology (FTIR) spectroscopy uses infrared light to identify the chemical composition of a sample by analyzing its absorption pattern^7^, while second harmonic generation (SHG) microscopy offers high-resolution visualization of non-centrosymmetric structures such as collagen^8^. Beyond THG, stimulated Raman scattering (SRS) microscopy provides complementary chemical contrast by probing vibrational resonances with submicron resolution^9^. SRS directly maps biomolecular species—such as lipids, proteins, and metabolites—without labels. Recent advances in multiplexed and hyperspectral SRS now enable simultaneous visualization of multiple molecular components, offering a richer biochemical context for interpreting tissue architecture. Nevertheless, all these pure optical technologies suffer from limited penetration or lack sensitivity to microstructural orientation. Overcoming these constraints requires a system that merges molecular specificity with directional structural information—a milestone now reached by Park et al. in *Light: Science & Applications*^10^.

In fact, photoacoustic microscopy (PAM) has emerged as an alternative, converting absorbed optical energy into ultrasonic readouts^11–16^. Exploiting endogenous chromophores, PAM provides intrinsic structural and functional contrast without labels^17–19^. For histopathology, ultraviolet (UV)-PAM highlights cell nuclei structures via DNA/RNA chromophore excitation^20–23^. While UV-PAM excels in nuclear contrast, mid-infrared (MIR)-PAM enables analytical histology while maintaining high spectral resolution^24,25^. MIR-PAM bridges the gap between morphology and molecular composition, capable of imaging proteins, lipids and carbohydrates^24,26–28^.

In 2018, Yuan Qu et al. proposed dichroism-sensitive photoacoustic computed tomography (DS-PACT), revealing that many tissues exhibit optical absorption anisotropy, and that the anisotropy could be imaged by modulating the polarization of the illuminating light in photoacoustic imaging^29^, and subsequent extensions were implemented to PAM systems^30,31^. Illuminating tissues with linearly polarized light at various polarization angles results in measurable changes in photoacoustic signals. These signals are then used to calculate the amplitude of the dichroism and the orientation of the optic axis.

Building on these polarization-resolved PA systems, Park et al. now report a mid-infrared dichroism-sensitive photoacoustic microscopy (MIR-DS-PAM) system that uses mid-infrared (MIR) light to simultaneously quantifies a tissue’s protein content and images its microstructural organization, enabling both compositional and structural contrast (Fig. 1)^10^. Using engineered heart tissues (EHTs), the authors showed that MIR-DS-PAM selectively visualizes protein-rich regions of the extracellular matrix (ECM) and tracks changes in fibrotic alignment during tissue maturation. By analyzing the degree and orientation of linear dichroism (DoLD and AoLD), the authors quantified the organization and uniformity of ECM fibers, confirming that MIR-DS-PAM effectively captured structural remodeling during tissue maturation and fibrotic progression. Compared to conventional fluorescence microscopy, MIR-DS-PAM offered comparable orientation mapping without the need for staining or antibody labeling, enabling direct histostructural evaluation. The system further distinguished between two fibrotic models, cell-induced and drug-induced fibrosis, revealing unique molecular and structural phenotypes consistent with immunofluorescence results. This represents a long-sought capability in label-free pathology—linking molecular specificity with structural clarity in a single imaging framework. This works exemplifies a growing paradigm shift in histopathology: from staining-dependent imaging toward quantitative, label-free molecular readouts. Techniques such as digital virtual staining demonstrated by Yoon et al.^32^ and polarization sensitive optics^33^ have hinted at this transition, but MIR-DS-PAM brings both biochemical specificity and physical anisotropy into a unified analytical framework.

**Fig. 1:**
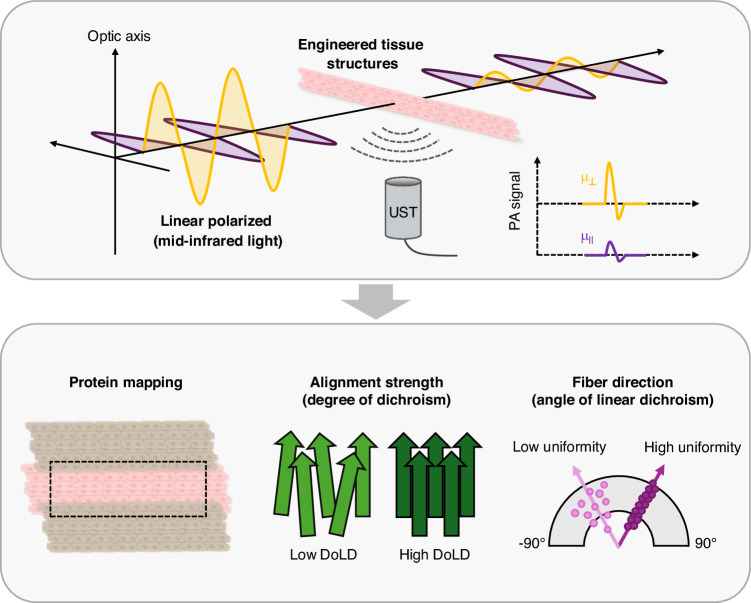
Mid-infrared dichroism-sensitive photoacoustic microscopy (MIR-DS-PAM) for label-free mapping of tissue composition and fiber alignment. Mid-infrared pulses at the amide-I vibrational band generate photoacoustic signals from protein-rich regions of the extracellular matrix. By acquiring images at four incident polarization angles, MIR-DS-PAM analyzes polarization-dependent absorption arising from anisotropic molecular organization. The resulting dichroism metrics—the degree of linear dichroism (DoLD) and the angle of linear dichroism (AoLD)—quantitatively map fiber alignment and orientation. Applied to engineered heart tissues, this dual-contrast method visualizes protein content while resolving microstructural organization, enabling label-free assessment of tissue maturation and fibrotic remodeling

The integration of mid-infrared molecular specificity with polarization-sensitive photoacoustic microscopy marks a major advance toward analytical, label-free histopathology. The authors elegantly demonstrate a framework that bridges molecular composition and structural anisotropy, setting a new benchmark for fibrosis and tissue engineering research. By directly quantifying molecular anisotropy, MIR-DS-PAM may enable new metrics of tissue remodeling and fibrosis staging- parameters that traditional histology can only infer indirectly. Nevertheless, several limitations remain. The spatial resolution is currently constrained by the diffraction limit of MIR light with long wavelengths, which notably restricts the visualization of individual fiber features. Additionally, the system operates at a single excitation wavelength, limiting its molecular specificity to protein-associated absorption bands. The work limits itself to end-point analysis as well, focusing on imaging fixed EHTs at specific day points. Despite these challenges, the work establishes a clear pathway forward: incorporation of resolution enhancement methods^34^, multi-wavelength excitation imaging systems^35^, and extending MIR-DS-PAM from in-vitro engineered tissues to preclinical and clinical biopsy models. This dual-contrast framework could redefine histopathological workflows, providing real-time, quantitative, and label-free analysis of tissue remodeling and disease progression. By revealing molecular orientation without labeling, MIR-DS-PAM may ultimately redefine how structural pathologies are visualized and classified, marking a shift from descriptive imaging to quantitative, physics-based pathology.
